# Supplementary material for: Characterization of Lung Microbiomes in Pneumonic Hu Sheep Using Culture Technique and 16S rRNA Gene Sequencing
Source: Animals (Basel). 2023 Aug 30;13(17):2763. doi: 10.3390/ani13172763 (PMC10486422; doi:10.3390/ani13172763)
Supplement: Supplementary file 1 [file animals-13-02763-s001.zip › Supplementary Figure S2.pdf]

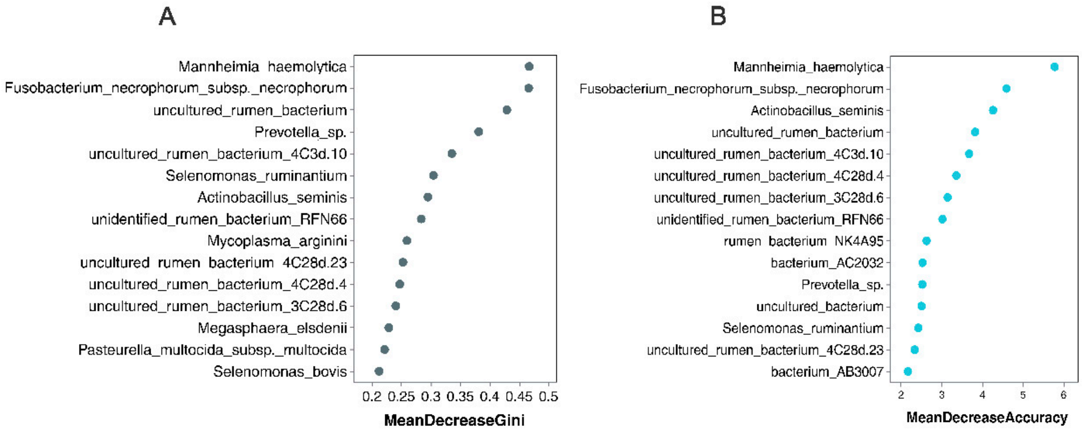

Supplementary Figure S2 The lung microbiota signature of sheep with varying degrees of pneumonia was determined using Random Forest analysis (95% correct, in Species level). (A) The mean decrease gini. (B) The mean decrease accuracy
