# Supplementary material for: Characterization of Lung Microbiomes in Pneumonic Hu Sheep Using Culture Technique and 16S rRNA Gene Sequencing
Source: Animals (Basel). 2023 Aug 30;13(17):2763. doi: 10.3390/ani13172763 (PMC10486422; doi:10.3390/ani13172763)
Supplement: Supplementary file 1 [file animals-13-02763-s001.zip › Supplementary Table S1.pdf]

Supplementary Table S1. Clinical assessment report from the healthy and pneumonia Hu sheep.

Sheep ear tag ID	Analysis ID	Male or Female	Age (days)	Weight (kg)	Eye score	Nasal score	Rectal temp	Cough	Moribund	Respiratory distress	Respiratory score
F2204317	H	F	126	29.4	0	0	38.2	0	-	-	1
F14	H2	F	151	34.2	0	1	38.3	0	-	-	0
F34442	H3	F	138	31.8	0	0	38.4	0	-	-	1
F33611	H4	M	121	28.5	0	0	39.0	0	-	-	0
F1301935	H5	M	142	31.5	1	0	38.5	0	-	-	0
F13	H6	M	146	30.9	0	0	39.0	0	-	-	1
F2205077	M1	M	120	28.3	1	1	39.9	1	-	-	2
F2207232	M2	F	136	32.9	1	1	40.4	1	-	-	3
FHY	M3	F	145	34.3	0	2	41.5	1	-	-	3
F40071	M4	M	137	30.1	0	1	40.6	1	-	-	3
F51583	M5	M	140	31.7	0	1	39.7	1	-	+	4
F37032	M6	M	157	29.5	0	1	39.4	1	-	-	2
F01847	S1	F	161	27.9	1	2	37.3	3	+	+	4
F11631	S2	M	153	25.8	1	2	36.5	2	+	+	4
F11908	S3	M	129	25.1	1	2	37.7	2	+	+	4
F12080	S4	M	137	24.7	1	1	37.5	3	+	+	4
F55597	S5	F	146	26.5	2	2	38.1	2	-	+	3
F2203601	S6	M	124	23.7	1	2	37.1	3	+	+	4

Eye, nasal, cough and respiratory score were classified referenced the Wisconsin sheep health scoring criteria (https://www.vetmed.wisc.edu/dms/fapm/fapmtools/8sheep/sheep_respiratory_scoring_chart.pdf).
