# Supplementary material for: Characterization of Lung Microbiomes in Pneumonic Hu Sheep Using Culture Technique and 16S rRNA Gene Sequencing
Source: Animals (Basel). 2023 Aug 30;13(17):2763. doi: 10.3390/ani13172763 (PMC10486422; doi:10.3390/ani13172763)
Supplement: Supplementary file 1 [file animals-13-02763-s001.zip › Supplementary Table S2.pdf]

Supplementary Table S2: Description of the lungs from the clinically healthy calves observed following slaughter.

Sheep ear tag ID	Moribund	Adhesion of the lung to the thoracic wall.	Description of lungs following slaughter
F2204317-H1	No	No	Clinically healthy lungs
F14-H2	No	No	Clinically healthy lungs
F34442-H3	No	No	Clinically healthy lungs
F33611-H4	No	No	Lesions present on a small part of the right middle lobe
F1301935-H5	No	No	Small lesions present on one cranial lobe
F13-H6	No	No	Clinically healthy lungs
F2205077-M1	No	No	Lesions present on both cranial lobes
F2207232-M2	No	No	Small lesions present on middle lobes in right Lung
FHY-M3	No	No	Lesions present on left lung
F40071-M4	No	Yes	Small lesions present on middle lobes in left Lung
F51583-M5	No	No	Small lesions present on middle lobes in left Lung
F37032-M6	No	No	Lesions present on a small part of the left middle lobe
F01847-S1	Yes	Yes	Lesions present on left lung
F11631-S2	Yes	Yes	Severe lesions present on both cranial lobes and lesions present on middle lobe
F11908-S3	Yes	Yes	Severe lesions present on both cranial lobes and lesions present on middle lobe
F12080-S4	Yes	No	Severe lesions present on both cranial lobes and lesions present on middle lobe
F55597-S5	Yes	Yes	Lesions present on both cranial lobes
F2203601-S6	Yes	Yes	Severe lesions present on both cranial lobes and lesions present on middle lobe

Note: Lesions were defined as macroscopic consolidation or abscessation of lung tissue.
