# Supplementary material for: Characterization of Lung Microbiomes in Pneumonic Hu Sheep Using Culture Technique and 16S rRNA Gene Sequencing
Source: Animals (Basel). 2023 Aug 30;13(17):2763. doi: 10.3390/ani13172763 (PMC10486422; doi:10.3390/ani13172763)
Supplement: Supplementary file 1 [file animals-13-02763-s001.zip › Supplementary Table S3.pdf]

**Supplementary Table S3. Significant difference in community structure among three groups.**

| level | dist | test | sub-condition1 vs sub-condition2 | Df | SumsOfSqs | MeanSqs | F.Model | R2 | *P*-value |
|---|---|---|---|---|---|---|---|---|---|
| Genus | bray | adonis | Health vs Moderate | 1 | 0.788 | 0.788 | 6.33 | 0.388 | 1.00E-03 |
| Genus | bray | adonis | Health vs Severe | 1 | 0.507 | 0.507 | 5.08 | 0.337 | 3.00E-03 |
| Genus | bray | adonis | Moderate vs Severe | 1 | 1.14 | 1.14 | 12.5 | 0.556 | 3.00E-03 |
| Genus | bray | adonis | all_groups | 2 | 1.62 | 0.811 | 7.72 | 0.507 | 1.00E-03 |
