# Supplementary material for: Characterization of Lung Microbiomes in Pneumonic Hu Sheep Using Culture Technique and 16S rRNA Gene Sequencing
Source: Animals (Basel). 2023 Aug 30;13(17):2763. doi: 10.3390/ani13172763 (PMC10486422; doi:10.3390/ani13172763)
Supplement: Supplementary file 1 [file animals-13-02763-s001.zip › Supplementary Table S4.pdf]

**Supplementary Table S4. Statistical comparison of differential taxa among the sheep with asymptomatic, moderately symptomatic, and severely symptomatic pneumonia. All the data are represented as mean ± SD.**

| Taxa (Genus) | Health_ Average (%) | Moderate_ Average (%) | Severe_ Average (%) | p-value |
|---|---|---|---|---|
| **Mannheimia** | **12.76757** | **60.53734** | **11.57785** | **0.003591003** |
| **Fusobacterium** | **14.23691** | **6.769017** | **44.57451** | **0.003591003** |
| **Bacteroides** | **8.352825** | **1.713873** | **19.70989** | **0.003591003** |
| Pasteurella | 12.06976 | 7.750702 | 2.374826 | 0.324638288 |
| (Unassigned) | 9.971365 | 5.724249 | 2.636018 | 0.324638288 |
| Prevotella_1 | 7.944912 | 1.045831 | 1.124862 | 0.163223951 |
| Mycoplasma | 2.416291 | 4.808128 | 2.766791 | 0.735462785 |
| Filobacterium | 1.466857 | 0.062374 | 5.147288 | 0.394490896 |
| Bacillus | 4.591945 | 0.059185 | 1.004012 | 0.394490896 |
| Histophilus | 0.707026 | 2.967736 | 0.180389 | 0.464903274 |
| Helcococcus | 1.097219 | 1.377194 | 1.331831 | 0.944523342 |
| **Prevotella_7** | **3.013453** | **0.045009** | **0.127938** | **0.04296142** |
| **Succiniclasticum** | **2.451022** | **0.19173** | **0.27395** | **0.044379594** |
| Rikenellaceae_RC9_gut_group | 1.429645 | 0.453276 | 0.351918 | 0.16640428 |
| Enterococcus | 1.227992 | 0.045363 | 0.331363 | 0.169766581 |
| **Bibersteinia** | **0.069462** | **1.171642** | **0.216892** | **0.003591003** |
| Streptococcus | 0.708089 | 0.25623 | 0.228942 | 0.39479873 |
| Campylobacter | 0.45115 | 0.139633 | 0.514233 | 0.264640618 |
| Moraxella | 0.586176 | 0.352981 | 0.164795 | 0.487140228 |
| Acinetobacter | 0.74282 | 0.041819 | 0.302302 | 0.225220047 |
| Fibrobacter | 0.915058 | 0.034377 | 0.086828 | 0.16640428 |
| Chryseobacterium | 0.429886 | 0.419254 | 0.138216 | 0.735259832 |
| Porphyromonas | 0.43024 | 0.052451 | 0.398344 | 0.324638288 |
| Treponema_2 | 0.603896 | 0.142468 | 0.118724 | 0.113931028 |
| Ruminococcaceae_UCG-005 | 0.548964 | 0.085056 | 0.135026 | 0.324638288 |
| Psychrobacter | 0.324629 | 0.174364 | 0.183224 | 0.735462785 |
| Lachnospiraceae_NK3A20_group | 0.483754 | 0.090372 | 0.10313 | 0.070655445 |
| Megasphaera | 0.609212 | 0.00567 | 0.02091 | 0.394490896 |
| Christensenellaceae_R-7_group | 0.307618 | 0.107737 | 0.134672 | 0.17135355 |
| uncultured | 0.209095 | 0.124394 | 0.212994 | 0.394490896 |
| **Pseudomonas** | **0.060602** | **0.359715** | **0.109509** | **0.045852117** |
| Roseburia | 0.396927 | 0.038275 | 0.02339 | 0.263546356 |
| Prevotellaceae_UCG-001 | 0.26828 | 0.074778 | 0.099586 | 0.260039921 |

| | | | | |
|---|---|---|---|---|
| **Peptoniphilus** | **0.090726** | **0.041819** | **0.308681** | **0.040976099** |
| Saccharofermentans | 0.262964 | 0.040047 | 0.125457 | 0.394490896 |
| Rothia | 0.306909 | 0.031896 | 0.075487 | 0.152197905 |
| Ruminococcus_1 | 0.325338 | 0.015594 | 0.046781 | 0.062858718 |
| Paracoccus | 0.294151 | 0.071234 | 0.020201 | 0.487140228 |
| **Escherichia-Shigella** | **0.001772** | **0.29486** | **0.082929** | **0.007043256** |
| **Mogibacterium** | **0.255522** | **0.070171** | **0.0443** | **0.02247347** |
| Ruminococcaceae_UCG-014 | 0.230005 | 0.044654 | 0.060248 | 0.048717079 |
| Prevotellaceae_UCG-003 | 0.145658 | 0.061665 | 0.12085 | 0.48729788 |
| Ruminococcus_2 | 0.240637 | 0.03544 | 0.038629 | 0.168131894 |
| Clostridium_sensu_stricto_1 | 0.09994 | 0.123685 | 0.090017 | 0.903986257 |
| Alloprevotella | 0.231422 | 0.021973 | 0.05883 | 0.324638288 |
| Terrisporobacter | 0 | 0.193147 | 0.118724 | 0.152197905 |
| p-1088-a5_gut_group | 0.239219 | 0.034377 | 0.032959 | 0.394490896 |
| Ruminococcaceae_NK4A214_group | 0.152391 | 0.046072 | 0.059539 | 0.271625453 |
| Succinivibrionaceae_UCG-001 | 0.212994 | 0.007088 | 0.015948 | 0.263546356 |
| Acetitomaculum | 0.159479 | 0.038629 | 0.028706 | 0.299956738 |
| Lysinibacillus | 0.163378 | 0.025871 | 0.036857 | 0.394490896 |
| Delftia | 0.089663 | 0.096396 | 0.03544 | 0.704809767 |
| Sphaerochaeta | 0.185705 | 0.014885 | 0.018429 | 0.394490896 |
| Family_XIII_AD3011_group | 0.124748 | 0.046781 | 0.045363 | 0.148734059 |
| Selenomonas_1 | 0.187831 | 0.008506 | 0.007088 | 0.424888837 |
| Lachnoclostridium_1 | 0.178617 | 0.003898 | 0.009569 | 0.324638288 |
| Prevotellaceae_Ga6A1_group | 0.166213 | 0.003898 | 0.010278 | 0.394490896 |
| Selenomonas | 0.149911 | 0.006379 | 0.018074 | 0.070851802 |
| Succinivibrio | 0.151683 | 0.004607 | 0.013113 | 0.438715827 |
| Pyramidobacter | 0.157707 | 0.005316 | 0.00567 | 0.213116767 |
| Serratia | 0.041465 | 0.01453 | 0.108092 | 0.394490896 |
| Syntrophococcus | 0.14176 | 0.009214 | 0.009569 | 0.089943414 |
| Ruminococcaceae_UCG-002 | 0.086828 | 0.046072 | 0.02658 | 0.394490896 |
| Veillonellaceae_UCG-001 | 0.075487 | 0.036503 | 0.039338 | 0.735462785 |
